# Supplementary material for: Association between oral microbiome and seven types of cancers in East Asian population: a two-sample Mendelian randomization analysis
Source: Front Mol Biosci. 2023 Nov 21;10:1327893. doi: 10.3389/fmolb.2023.1327893 (PMC10702768; doi:10.3389/fmolb.2023.1327893)
Supplement: Supplementary file 1 [file DataSheet1.ZIP › Supplementary Materials/MR plots of saliva/saliva═╝/colorectal cancer/pheno.3376_to_colorectal cancer_leave_one_out.pdf]

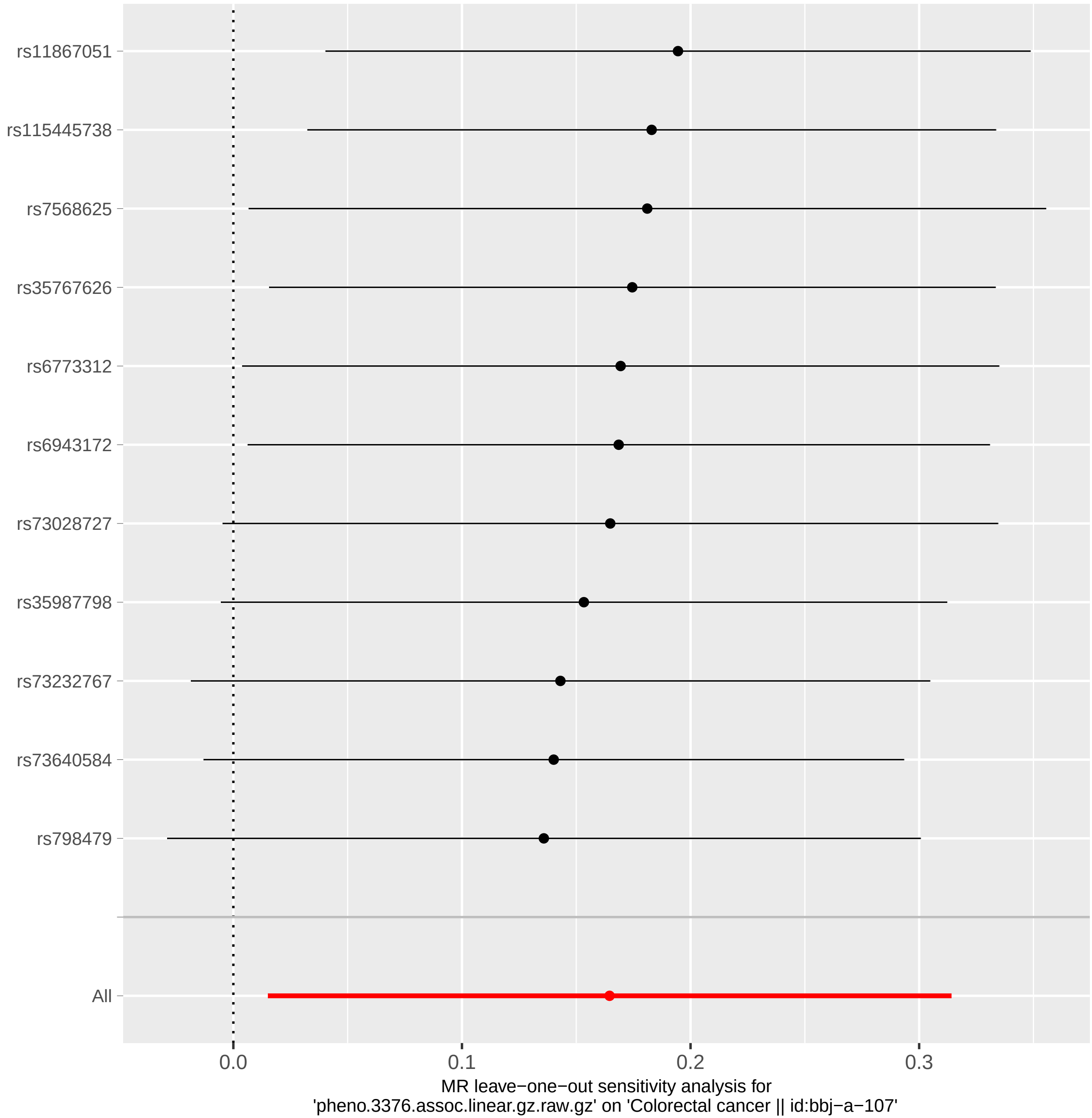
rs11867051
rs115445738
rs7568625
rs35767626
rs6773312
rs6943172
rs73028727
rs35987798
rs73232767
rs73640584
rs798479
All
0.0
0.1
0.2
0.3
MR leave−one−out sensitivity analysis for
'pheno.3376.assoc.linear.gz.raw.gz' on 'Colorectal cancer || id:bbj−a−107'
